# Is negative parenting always harmful? The differential effects of parental rejection and parental overprotection on proactive personality in young adults

**DOI:** 10.3389/fpsyg.2026.1798297

**Published:** 2026-05-28

**Authors:** Xin Zhou, Kangsheng Tao, Mi Zhang, Jin Huang

**Affiliations:** 1 College of Humanities and Education, Enshi Polytechnic, Enshi, China; 2 School of Ethnology and Sociology, South-Central Minzu University, Wuhan, China; 3 Business School, Enshi Polytechnic, Enshi, China; 4 Department of Anesthesiology, Zhongnan Hospital of Wuhan University, Wuhan, China; 5 Personnel Division, Enshi Polytechnic, Enshi, China

**Keywords:** parental overprotection, parental rejection, positive coping strategies, proactive personality, teacher-student support

## Abstract

The present study is anchored in the theoretical framework of ecological systems. Its objective is to investigate the underlying mechanisms through which negative parenting styles exhibited by parents influence the proactive personality of their offspring. The study will focus on the mediating effects of positive coping strategies and the moderating influences of teacher-student support. This study draws on survey data collected from a sample of 695 Chinese university students to empirically test the proposed hypotheses. The results demonstrated that: Initial findings indicated that parental rejection was positively associated with the development of a proactive personality in young adults, while parental overprotection was negatively associated with this personality. Secondly, the study established that positive coping strategies play a mediating role between the two types of parenting styles and proactive personality. Thirdly, it was demonstrated that higher levels of teacher–student support strengthen the mediating role of positive coping strategies in the relationship between parental rejection and young adults’ proactive personality. However, the moderating effect of the variable on parental overprotection was found to be non-significant. This study identifies distinct pathways through which different negative parenting styles shape proactive personality, and provides a theoretical basis and practical enlightenment for the collaborative intervention of family education and school support systems.

## 1 Introduction

Proactive personality refers to a relatively stable psychological tendency in which individuals adopt proactive attitudes and actions to influence or change their own circumstances and environment (Bateman and Crant, 1993; Kumar and Shukla, 2022). As an important trait for predicting career success, mental health, and adaptability, the formation mechanism of proactive personality has drawn attention from multiple disciplines, including psychology, education, and sociology. Existing research has revealed that it is influenced by factors at multiple levels, such as work contexts (Crant, 1995; Karimi et al., 2022), individual characteristics (Crant, 2000; McCormick et al., 2019), as well as family (Cunningham and de la Rosa, 2008; Preston and Salim, 2019; Zhang et al., 2022) and school environments (Gao et al., 2020; Ozkurt and Alpay, 2018; Wen et al., 2022).

Within the family system, parenting styles serve as a core mechanism of individual socialization and play a pivotal role in the development of proactive personality in young adults. Parenting styles refer to the relatively stable patterns of behaviors and emotional attitudes that parents display during the childrearing process (Shloim et al., 2015; Garcia et al., 2020). These patterns generally encompass dimensions such as rejection, emotional warmth, and overprotection (Hou et al., 2020; Khalid et al., 2019). Rejection and overprotection are regarded as archetypal negative parenting styles that may pose risk factors for young adults’ development of proactivity. However, extant studies have reached inconsistent conclusions regarding the effects of these two styles, and these effects have been shown to be moderated by factors such as cultural background and parental roles (Preston and Salim, 2019; Hong and Dyakov, 2021). In addressing the issue of parental roles, it is evident that both fathers and mothers play both overlapping and distinct functional roles in the process of children’s socialization (Zhang et al., 2024). According to activation relationship theory, the primary function of the mother is to provide emotional security and the foundations for attachment, whereas the father’s role is to promote his children’s independent development through encouraging risk-taking and stimulating exploration, thereby serving the role of “activator” (Paquette and StGeorge, 2023). Consequently, the nature and influence of paternal and maternal rejection or overprotection may differ (Kochanska et al., 2026). However, in the case of young people, the parenting behaviors of both parents do not operate in isolation; rather, they converge into an overall family parenting climate that systematically shapes personality development (de Mendonça et al., 2011).

Although existing research has confirmed the association between negative parenting styles and proactive personality, most studies remain at the descriptive level of their correlation, or merely examine a single mediating variable (such as self-efficacy), failing to systematically reveal the complex psychological processes underlying this relationship. In addition, extant literature demonstrates a greater focus on the overall type or single dimension of parenting style. Furthermore, the text lacks a differentiated comparison and theoretical interpretation of the influence path of different negative parenting dimensions. Finally, it examines the regulatory effect of external protective factors from the perspective of multi-system interaction to a lesser extent. According to Bronfenbrenner’s ecological systems theory (Bronfenbrenner and Morris, 2006), individual development is nested within mutually influencing environmental systems. Consequently, a combination of individual psychological processes and situational factors is required to elucidate the mechanism of personality formation.

In consideration of the aforementioned limitations, the present study proposes the introduction of two variables: namely, positive coping strategies and teacher-student support. The objective of this study is to establish a moderated mediation model in order to enhance the understanding of the underlying mechanisms through which negative parenting styles influence proactive personality. The term “coping strategies” refers to the strategies an individual employs in response to pressure. These coping strategies can be categorized as either positive or negative, as posited by Folkman and Lazarus (1988) and further elaborated by Lipp and O’Brien (2022). Research indicates that negative parenting practices may diminish an individual’s propensity to adopt positive coping mechanisms (Rawlings et al., 2022; Cheng et al., 2022), thereby impeding the development of their proactive personality. Meanwhile, as an important microsystems, the teacher-student support provided by schools may play a more profound moderating role. High levels of teacher support not only enhance young adults’ autonomy and psychological resources through emotional care, competence recognition, and resource provision (Eccles and Roeser, 2011; Chen, 2008), but also change their cognitive appraisal of family stress, prompting them to reframe originally threatening stress as manageable challenges, thereby making them more inclined to adopt positive coping strategies (Kit et al., 2022). When young people perceive teachers’ emotional support, their academic self-efficacy improves, and their educational hope strengthens; this positive cognitive state further reinforces their ability to transform external support into internal motivation (Guo et al., 2025). Therefore, teacher-student support may function through two pathways: buffering the adverse effects of negative parenting, or reinforcing the protective effects of positive coping strategies. However, this moderating pathway has not been sufficiently examined in existing research.

In summary, the present study is founded upon the ecological systems theory, with a focus on the two types of negative parenting dimensions of “rejection” and “overprotection.” The aim is to achieve the following research objectives through a regulated mediation model: The present study sets out to achieve three objectives. Firstly, it will compare the differences in the effects of two kinds of negative parenting styles on young adults’ initiative personality. Secondly, it will test the mediating role of positive coping strategies in the relationship between the two. Thirdly, it will investigate the moderating effect of teacher–student support in the mediating path. The findings of this research are expected to contribute to a more comprehensive understanding of the differential mechanisms through which negative parenting affects proactive personality in a theoretical context. Furthermore, these results are anticipated to provide a practical foundation for family and educational settings to promote the development of youth initiative in a tangible manner.

## 2 Theory and hypothesis

In accordance with the research questions posed in the Introduction section, this section will construct a moderated mediation model based on ecological systems theory as the theoretical framework for this study, and propose specific research hypotheses accordingly.

### 2.1 Ecological systems theory

The present study employs the ecological systems theory (Bronfenbrenner, 1979) as a core theoretical framework to elucidate the complex impact of parental negative parenting styles on proactive personality in young adults. According to this theory, individual development is embedded within a set of interconnected environmental systems, and a thorough understanding of developmental trajectories requires examining the interactions among these systems (Ceci, 2006; Ozaki et al., 2020; Osorio and González-Cámara, 2016). This theoretical perspective is among the most frequently cited in the field of developmental psychology (Crawford, 2020; Yu et al., 2021), and has been extensively applied to research on individual development, as well as to the study of how family and school environments interact to influence individual development (Dobson and Douglas, 2020; El Zaatari and Maalouf, 2022). This renders it especially well-suited for the analysis of research problems that span family and school microsystems.

The present study adopts a family microsystems perspective, positing that the negative parenting style of parents constitutes the core risk factor of this system, exerting a direct influence on the establishment of proactive personality in young adults. Positive coping strategies, defined as an individual’s cognitive and behavioral strategy in the face of stress (Folkman and Lazarus, 1988), can be conceptualized as a key proximal process connecting the family microsystems with individual psychological outcomes (proactive personality). The ecological systems theory posits the occurrence of interactions between disparate microsystems. The interaction of teacher–student support in school microsystems with home microsystems can be conceptualized as a critical external resource. Specifically, high-level support from teachers and students may act as a protective factor, buffering the adverse effects of negative parenting on young adults’ coping strategies or directly enhancing their coping effectiveness. This underscores the profound influence of intersystem interactions on the developmental trajectories of individuals.

The present study’s theoretical framework is thus guided by the theory of ecological systems. The framework’s design enables the clear demonstration of the influence of parental negative parenting styles (family microsystems) on the development of proactive personality through the shaping of young adults’ coping strategies (individual proximal processes). The efficacy of this mediating pathway is moderated by teacher-student support within school microsystems. This moderated mediation model provides a systematic theoretical perspective on the formation mechanism of proactive personality in young adults, reflecting the interactions between different systems.

### 2.2 Research hypothesis

**Figure 1:**
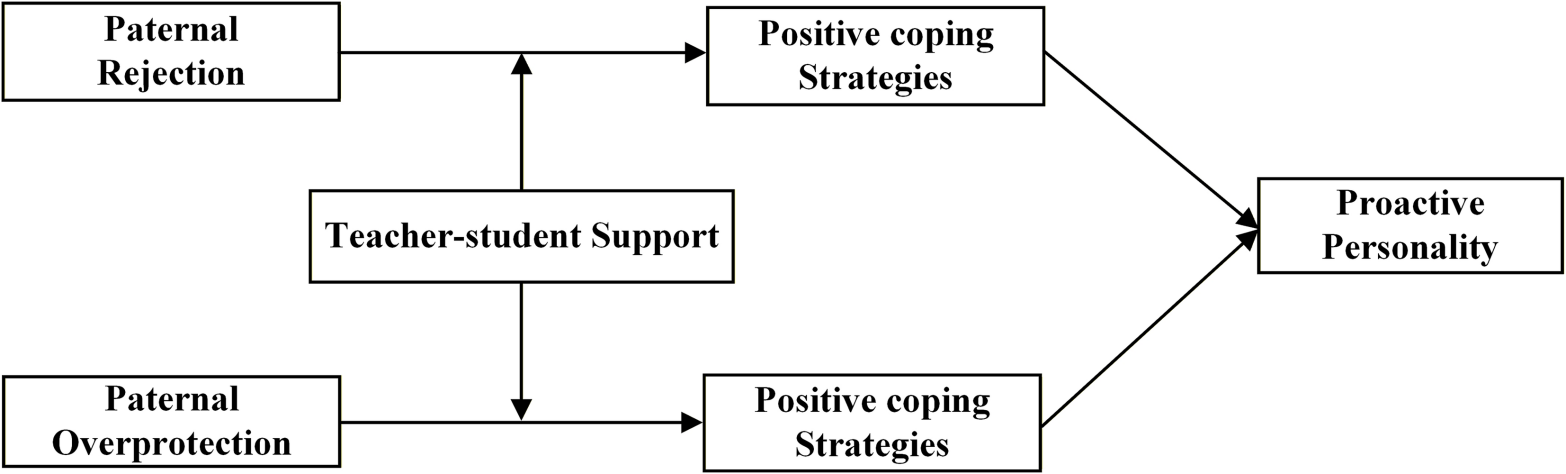
Theoretical model.

The present study proposes a moderated mediation model to systematically examine the relationships among parental negative parenting styles, positive coping strategies, teacher-student support, and proactive personality in young adults. The model under consideration is predicated on the tenets of ecological systems theory. The theoretical model is illustrated in Figure 1.

#### 2.2.1 Negative parenting styles and proactive personality of young adults

The family, as a microsystems of individual socialization, plays a foundational role in shaping young adults’ behavioral habits, values, and personality (Zhang and Wang, 2020). In this system, parental upbringing as a core element may exert a more complex influence on the individual than traditional cognition. Despite the prevalence of the notion that rejection parenting constitutes a risk factor, characterized by parental emotional apathy, frequent criticism, and strict discipline (Mak et al., 2020), from the perspective of adversity growth, this specific stressor has the capacity to stimulate individuals’ psychological resilience and motivation for proactive change in specific contexts (Stern and Zevon, 1990; Linley and Joseph, 2004; Joseph and Linley, 2006).

Specifically, in the case of young adults who have experienced protracted adversity, characterized by an absence of emotional support and a curtailing of autonomy, this deleterious experience has the potential to engender a disruption of their original core beliefs. In order to cope with the crisis of belief and adapt to the environment, individuals are compelled to explore new possibilities, namely finding ways to meet their own developmental needs outside the family system. This process of exploration has been shown to result in the development of a series of compensatory proactive strategies (Roepke and Seligman, 2015; Siebert et al., 2020). The “adversity” engendered by rejection parenting has been demonstrated to have a non-unilateral effect on development; rather, it has been shown to act as a “push” that prompts individuals to acquire personality traits of independence, active exploration, and problem-solving earlier outside the family system. This approach is indicative of an adaptive strategy for managing family-related stress (Camisasca et al., 2022; Di Giunta et al., 2022). From a positive youth development perspective, this pathway of “adversity-induced proactivity” is not an isolated psychological compensation; rather, it reflects the bidirectional interplay between individual strengths and contextual resources when the family system is functioning inadequately (Gomez-Baya et al., 2025).

It is noteworthy that paternal rejection and maternal rejection may carry different psychological meanings. Empirical research suggests that fathers play a unique “secure base” role in encouraging children to explore the external world independently, and that paternal support is even stronger than maternal support in predicting young adults’ positive emotions (Peng et al., 2024). Consequently, paternal rejection may be cognitively constructed by children as a denial and hindrance to their independent competence development, thereby eliciting a stronger motivation for “adversity-driven growth” in order to prove their own worth; whereas maternal rejection tends more to undermine the foundation of emotional security. Although this role-differentiation perspective aligns more closely with a Western individualistic context, its underlying mechanism also gains support from cultural logic in the Chinese collectivist cultural setting. Specifically, the Chinese family is regarded as a highly integrated system, characterized by close bonds built on mutual responsibilities (Liu, 2012). Confucian meritocratic cultural scripts such as “adversity builds character” and “only by enduring the hardest hardships can one rise above others” provide legitimate resources for young people to interpret family rejection as a form of stressful yet motivating developmental impetus. Under the combined influence of tight family bonds and a sense of moral obligation, such pressure-laden expectations are more readily internalized as self-demands, transforming the alienation caused by rejection into a drive to prove one’s worth in broader social systems. Moreover, the interdependent self-construal prevalent among Chinese youth may further reinforce this process: when individuals define the self as closely connected to the family, family rejection may, due to unmet relatedness needs, activate an even stronger achievement motivation, with the aim of restoring or demonstrating that connection through social recognition (Chen et al., 2022). Based on this overall logic of “stress-activated initiative,” the present study proposes the following hypotheses:

*Hypothesis 1a*: Parental rejection positively affects young adult’s proactive personality

Parents’ overprotective parenting style is manifested in parents’ excessive intervention, excessive control over their children’s lives, and the tendency to solve problems for their children to avoid risks (Spokas and Heimberg, 2009; Moreira et al., 2021). Although this kind of parenting method reduces the setbacks faced by children in the short term, in the long run, it substantially deprives children of the opportunity to accumulate experience and develop abilities through independent exploration (Ungar, 2009). It is evident that young adults who have been in such an environment for a protracted period of time, due to the abridgement of decision-making power and the paucity of failure experience, find it challenging to cultivate intrinsic motivation and a sense of responsibility for their own behavior. Consequently, they are susceptible to forming a dependence on external guidance and demonstrate characteristics of evading challenges, as well as exhibiting a languid development of autonomous decision-making ability (Gere et al., 2012; Bruysters and Pilkington, 2023). These characteristics are in direct opposition to the characteristics of active exploration and seizing opportunities required by a proactive personality.

Viewing this further from the family systems perspective, the overall climate of overprotection may simultaneously inhibit the distinct developmental functions of fathers and mothers. According to activation relationship theory, the role of the father is to promote children’s independent development by encouraging risk-taking and autonomous decision-making, while the mother’s role is to provide emotional support and cognitive guidance, thus representing complementary role functions (Paquette and StGeorge, 2023). In circumstances where systemic autonomy deprivation is present, fathers are rendered incapable of fostering independent development through the encouragement of exploration. Conversely, the overprotective behavior of mothers, owing to its inherently controlling nature, may prove deficient in providing adequate emotional security to facilitate children’s autonomous exploration. This dual functional deficiency leaves young people lacking necessary risk-taking and decision-making practice both within and outside the family unit, thereby seriously impeding the development of their proactive personality. The following assumptions are proposed by this study:

*Hypothesis 1b*: Parental overprotection negatively affects young adult’s proactive personality

#### 2.2.2 Negative parenting style, positive coping strategies, young adult’s proactive personality

In accordance with the theoretical framework of the ecological systems, individual factors are identified as the pivotal proximal process, thereby connecting the experience of the microsystems and the outcomes of individual development. As previously stated, parental rejection, in the capacity of a source of adversity pressure, has the capacity to exert a positive influence on the initiative personality of individuals by stimulating their adaptive changes. In this study, proactive personality is defined as an individual’s stable tendency to take initiative, accept responsibilities, and engage in knowledge exploration (Bertolino et al., 2011). The present study hypothesizes that positive coping strategies function as the pivotal intermediary mechanism in the process of “stress motivating initiative.”

The psychological and behavioral tendency of “exploring new possibilities”, which is a consequence of a rejective upbringing environment (Walsh, 2011), is reflected in the application of a series of positive coping strategies at the operational level. In order to seek recognition and value outside the family, young adults may take the initiative to develop efficient coping abilities such as solving problems independently and actively seeking external support (Yanez et al., 2011). The study also highlighted the importance of developmental initiatives in fostering healthy individual growth in the context of adversity (Nielsen et al., 2023). This kind of positive coping ability, stimulated and strengthened under pressure, constitutes the core psychological resource for individuals to deal with environmental challenges (Plews-Ogan et al., 2019). When these coping strategies are employed repeatedly and with success, the knowledge gained can be generalized to other areas of the individual’s life. This process has the potential to gradually engender an elevated level of exploration spirit and goal pursuit, as well as other stable characteristics. This process is regarded as a pivotal intermediate mechanism through which the phenomenon of rejection parenting may engender an active personality. The following assumptions are thus proposed by this study:

*Hypothesis 2a*: Positive coping strategies play a mediating role between parental rejection and young adult’s proactive personality

Within the microsystems of family dynamics, the phenomenon of parental overprotection is theorized as a manifestation of high control and intervention. The fundamental objective of the system is to deny children the chance to cultivate their abilities through autonomous exploration, unconventional decision-making, and the overcoming of challenges (Bruysters and Pilkington, 2023). This paucity of developmental experience may systematically impede the acquisition of positive coping strategies among young adults. As demonstrated by previous studies, overprotection has been associated with maladaptive schemata, including dependence and emotional deprivation (Spokas and Heimberg, 2009; Li et al., 2025). This phenomenon has been demonstrated to diminish an individual’s confidence and efficacy in taking initiative by reducing self-concept and reinforcing external control points. This suggests that outcomes are determined by external factors. The interaction of these changes in cognition and motivation gives rise to a deficiency in positive coping ability. Consequently, the practice of overprotective parenting has the potential to indirectly hinder the development of young adults’ proactive personalities by impeding the cultivation of effective coping mechanisms. The following assumptions are proposed by this study:

*Hypothesis 2b*: Positive coping strategies play a mediating role between parental overprotection and young adult’s proactive personality

#### 2.2.3 The moderating effect of teacher-student support on parental rejection and young adult’s proactive personality

For young adults who have experienced long-term parental rejection, the development of positive coping mechanisms is often impeded. However, teacher support, encompassing emotional care, academic guidance and value recognition, can function as an external compensatory resource, thereby effectively buffering the impact of a negative family environment (Ansong et al., 2017; Li C. et al., 2022; Li J. et al., 2022). A high level of such support has been shown to enhance youth’s sense of self-worth. This reinforcement enables them to preserve the psychological resources necessary for problem-solving and to actively seek help despite experiencing familial negation, thereby attenuating the negative predictive effect of parental rejection on positive coping strategies (Klem and Connell, 2004).

Conversely, the provision of support by students has been demonstrated to engender an alternative positive social circle for young adults, characterised by companionship, cooperation and social acceptance (Tomé et al., 2014). The sense of belonging and successful experience gained in this environment have been shown to serve as a partial offset for the social anxiety and self-doubt that are often caused by family rejection (Busching and Krahé, 2020; Hoferichter et al., 2022). This, in turn, has been demonstrated to encourage individuals to adopt positive coping strategies, such as cooperation and disclosure, rather than avoidance and withdrawal when confronted with difficulties. Consequently, a high level of peer support is regarded as a protective factor, which is expected to weaken the negative correlation between parental rejection and positive coping strategies. In summary, teacher and student support is regarded as the key situational factor that can modify the relationship between parental rejection and positive coping strategies. The study thus proposes the following:

*Hypothesis 3a*: Teacher-student support plays a positive moderating role between parental rejection and positive coping strategies

Within the paradigm of parental overprotection, the central challenge often lies in the tendency for alternative decision-making and excessive intervention, with the aim of limiting young adults’ opportunities to develop independent coping abilities. In this context, teacher support can serve to counter the implicit message of “you cannot handle this independently” that overprotection may convey. This objective is realised through the provision of autonomy to students and the cultivation of their capacity for independent decision-making in academic and classroom contexts (Casas et al., 2013). The development of students’ sense of self-efficacy can be fostered by providing them with opportunities to make independent decisions in a safe environment and to achieve success in their academic pursuits. Consequently, such experiences can assist in the mitigation of the inhibitory effect of overprotection on positive coping strategies (Sadoughi and Hejazi, 2023). In a similar vein, students advocate for the establishment of an “autonomous practice field” for young adults, a field in which parents are unable to overstep their bounds by ensuring equal opportunities for cooperation and interaction with peers. In peer groups, the experience gained by individuals through autonomous negotiation and conflict resolution can compensate for a lack of social skills training due to excessive family protection, thereby enhancing their confidence in coping with social situations independently (Danielsen et al., 2010). The study thus proposes the following:

*Hypothesis 3b*: Teacher-Student support plays a negative moderating role between parental overprotection and positive coping strategies

#### 2.2.4 Teacher-student support moderated the mediating effect

As a pivotal protective factor, the provision of support by teachers and students may serve a moderating role in the mediating mechanism through which parental rejection exerts an influence on young adult’s proactive personality, by means of a positive coping strategies (Bokszczanin, 2012; Zhou et al., 2017).

A high level of teacher-student support may not only buffer the inhibitory effect of parental rejection on the development of young adult’s positive coping ability, but also provide them with key psychological resources to maintain their confidence in adopting positive strategies (Çevik and Yildiz, 2017); at the same time, it may also enhance the positive relationship between positive coping strategies and proactive personality, and further promote the transformation of adaptive behavior motivation by strengthening the successful experience of individuals (Zimmer-Gembeck and Locke, 2007). Regardless of the link along the aforementioned mediation path in which the moderating effect occurs, it is theoretically predicted that this effect will result in a stronger mediating effect of a positive coping strategies in contexts of higher support from teachers and students. This finding suggests that a robust school support system may play a pivotal role in promoting well-being, potentially mitigating the adverse impact of familial adversity by activating and fortifying the positive psychological mechanisms within individuals. It is anticipated and proposed by this study, based on the aforementioned theoretical derivation, that the following assumptions be made:

*Hypothesis 4a*: Teacher-student support moderates the mediating role of positive coping strategies. Specifically, the indirect effect of parental rejection on proactive initiative through positive coping strategies is enhanced when teacher-student support is higher.

In a similar vein, the present study hypothesized that teacher-student support would also moderate the indirect path through which parental overprotection influences proactive personality via positive coping strategies. It has been demonstrated that an excess of parental protection can impede the cultivation of positive coping ability by inhibiting the development of individual autonomy (Spada et al., 2012). Nonetheless, a high level of teacher-student support has been shown to engender a safe environment for young adults to make independent decisions and exercise their abilities. Such support can compensate, to a certain extent, for the lack of autonomy resulting from excessive familial protection, thereby weakening the inhibitory effect of overprotective parenting on positive coping ability. Correspondingly, greater levels of support from teachers and students are associated with a more substantial mitigation of the adverse impact of overprotective parenting, which in turn enables positive coping strategies to fulfil their adaptive function more effectively. The final result indicates that when teachers and students provide high levels of support, the indirect effect of parental overprotection on proactive personality through positive coping strategies is amplified. Based on this, the study proposes the following hypotheses:

*Hypothesis 4b*: Teacher-student support moderates the indirect effect of parental overprotection on proactive initiative through positive coping strategies. Specifically, this mediating effect strengthens as teacher-student support increases.

## 3 Methods and procedures

The present study adopts the questionnaire survey method and adheres rigorously to the norms of academic ethics. All procedures have been thoroughly reviewed and approved by the esteemed academic ethics committee of Enshi Polytechnic (Approval No. [2025]02). The data collection and handling procedures complied with applicable ethical standards.

### 3.1 Data collection

The data collection period is scheduled to occur from December 2025 to January 2026. Prior to the formal survey, the research team conducted a preliminary survey in two universities in China in December 2025. This survey was modified according to the subjects’ feedback, and the applicability of the questionnaire to different student groups was confirmed.

The participants of this study were young adults in the period of emerging adulthood, ranging in age from 18 to 25 years (Arnett, 2000). Early emerging adulthood (ages 18–21) is characterised by greater detachment from family dependence and active identity exploration, whereas late emerging adulthood (ages 22–25) involves the gradual establishment of stable commitments and transition to independent living (Tanner, 2006). To ensure adequate coverage of different developmental positions within this stage, and to avoid over-concentration of the sample at either end due to convenience sampling, both age ranges (18–21 and 22–25 years) were included in the sampling frame, thereby enhancing the overall representativeness of the emerging adult population (Delvecchio et al., 2023; Szwedo et al., 2017). The inclusion criteria were: (1) age between 18 and 25 years; (2) currently enrolled as an undergraduate student; (3) able to read and understand Chinese independently; and (4) voluntary participation with signed informed consent. The exclusion criteria were: (1) questionnaire completion rate below 90% (i.e., missing >10% of items); and (2) clearly invalid responses (e.g., selecting the same option for all items, contradictory answers to reverse-coded items, or completion time < 2 min).

Prior to the initiation of the formal investigation, the following institutions were approached for their insights on the aforementioned issues: South-Central Minzu University, Hubei Minzu University, Enshi Vocational and Technical College, and Enshi College. The investigation was conducted with the support of the relevant principals of these institutions. The survey instrument is in the form of an anonymous electronic questionnaire. This is compiled through a professional online survey platform Questionnaire Star and disseminated through WeChat, email and other channels. The questionnaire is divided into two sections. The first section collects demographic information, including gender, age, educational attainment and household registration. The second section comprises the student self-assessment scale of the core research variables. These include the following: parents’ negative parenting style, active personality, coping style and support from teachers and classmates. Before completing the questionnaire, all participants were fully informed about the study’s purpose, the confidentiality of their data, and the voluntary nature of their involvement. This information was conveyed to them via online instructions. A total of 792 questionnaires were distributed. After the initial collection, 718 questionnaires were preliminarily retained. To ensure data quality, responses were further screened according to the following criteria: inconsistent answers to reverse-worded items (*n* = 15), and completion time of less than 5 min, indicating potentially inattentive responding (*n* = 8). After excluding these 23 invalid responses, 695 questionnaires were deemed valid, resulting in an effective response rate of 87.8%.

As presented in Table 1, the sample consisted of 695 participants, predominantly female (76.0%) and aged 18–21 years (87.5%). Most were non-only children (78.6%) and came from two-parent households (85.6%). In terms of educational background, the majority were enrolled in junior college (56.8%) or undergraduate programs (32.9%), while only a small proportion were graduate students (0.7%). The educational attainment of the participants’ parents was generally modest, with 47.9% having completed junior high school or less and only 2.3% holding an undergraduate degree. Geographically, most participants resided in urban areas (86.0%). Household size was predominantly four or more members (76.7%), and 51.8% reported an yearly household income below ¥40,000. These demographic variables were included in the analyses as controls.

**Table 1:** Basic Information description.

Attributes	Items	Frequency	Percent (%)
Gender	Male	167	24.00
	Famale	528	76.00
Age	18–21	608	87.50
	22–25	87	12.50
Only child	Yes	149	21.40
	No	546	78.60
Single-parent family	Yes	101	14.41
	No	600	85.59
Respondent’s Educational Level	High school (vocational school)	66	9.50
	Junior college (including night universities)	395	56.80
	undergraduate	229	32.90
	Graduate students (master’s or doctoral)	5	0.70
Parents’ Educational Leve	Elementary school or below	177	25.50
	junior high school	333	47.90
	High school (vocational school)	126	18.10
	Junior college (including night universities)	42	6.00
	undergraduate	16	2.30
	Graduate students (master’s or doctoral)	1	0.10
Rural or urban residence	Urban	598	86.00
	Rural	97	14.00
Household size	Two or less	37	5.30
	Three	125	18.00
	Four	294	42.30
	Five or more	239	34.40
Yearly household income	Below 20,000 ¥	194	27.9
	20,000–30,000¥	166	23.9
	30,000–40,000¥	98	14.1
	40,000–50,000¥	76	10.9
	50,000–60,000¥	50	7.2
	60,000–70,000¥	44	6.3
	Over 80,000 ¥	67	9.6
Total		695	100.0

### 3.2 Measurements

The present study adopts the method of questionnaire survey, and all the tables are translated into Chinese following the classic back translation procedure of Brislin (1980), so as to ensure conceptual equivalence and linguistic appropriateness in the context of Chinese culture. The specific process is outlined as follows. Initially, a researcher with an international study background translated the English scale into the Chinese draft. Subsequently, an invitation was extended to a professor of domestic psychology to undertake the task of reviewing and correcting the initial draft, in addition to making adjustments to the expression of certain items in accordance with the received feedback. Finally, a researcher with an overseas background, who had no prior exposure to the original English version, performed back-translation of the final Chinese version into English. This back-translation was then compared with the original to ensure conceptual equivalence. In the survey, participants rated their level of agreement with each statement using a numerical scale. The scale of proactive personality was measured on a range from 1 to 5. The scale of negative parenting styles was measured on a range from 1 to 4. The scale of coping styles was measured on a range from 1 to 4. The scale of teacher-student support was measured on a range from 1 to 5.

#### 3.2.1 Negative parenting style

In this study, the Chinese version of the Simplified Parenting Style Questionnaire (s-EMBU-C) revised by Jiang and others (2010) was utilised to measure parents’ negative parenting styles. The original questionnaire contains multiple dimensions. In accordance with the theoretical framework, this study selected and integrated the two core dimensions of “rejection” and “overprotection,” which are closely related to negative parenting, with a view to more intensively reflecting the risk factors in parental rearing. The integrated scale contains 21 items, and is scored by Likert 4 points (1 = “never,” 4 = “always”). It is posited that an elevated score on the scale is indicative of a more pronounced degree of negative parenting. In the sample of this study, the two sub-dimensions of the integrated scale demonstrated adequate internal consistency: the Cronbach *α* coefficient of the parental refusal parenting style dimension was 0.871, and the parental overprotective parenting style dimension was 0.837.

#### 3.2.2 Proactive personality

The present study utilised the classic scale developed by Seibert et al. (1999) in order to measure the initiative personality of young adults. This one-dimensional scale measures an individual’s stable tendency to proactively change their environment and pursue long-term goals. It utilizes a 5-point Likert scoring system, ranging from 1 (“strongly disagree”) to 5 (“strongly agree”). The total score directly reflects the level of proactive personality, with higher scores indicating stronger proactive traits. In this study’s sample, the scale demonstrates excellent internal consistency, with an overall Cronbach’s *α* coefficient of 0.900.

#### 3.2.3 Coping strategies

The present study employs the Simple Coping Style Questionnaire, which was developed by Xie (1998) on the basis of Folkman and Lazarus’s theory. This scale is a mature tool in the Chinese context, which is used to measure habitual coping strategies of individuals in the face of stress events. The scale under consideration contains two relatively independent dimensions of positive coping and negative coping, with a total of 20 items. The employment of Likert 4-point scoring (1 = “never use,” 4 = “often use”) reveals a correlation between higher positive coping dimensions and the adoption of constructive strategies, such as confronting challenges directly and seeking assistance. Conversely, higher negative coping dimensions are associated with the adoption of non-constructive strategies, including avoidance and self-blame. In the present study’s sample, both dimensions of the scale show satisfactory internal consistency reliability, with the Cronbach’s α coefficient for the positive coping style dimension being 0.911.

#### 3.2.4 Teacher-student support

The two subscales of “school” and “friends” in the multidimensional students’ life satisfaction scale (MSLSS) developed by Huebner et al. (1994) were utilised in this study, and they were integrated and revised into a tool to measure teacher support and classmate support. The integrated scale comprises eight positive statements. The scale uses a 5-point Likert format ranging from 1 (“strongly disagree”) to 5 (“strongly agree”). It is evident that as the total score increases, the perceived level of support from teachers and classmates concomitantly rises. In the sample of this study, the overall Cronbach *α* coefficient of the integrated scale is 0.869, indicating that it has good internal consistency reliability.

#### 3.2.5 Control variable

As demonstrated in previous studies (Adedeji et al., 2022), young adult’s personal characteristics, including gender, age, and educational background, have been shown to have a significant impact on their proactive personality. Family environmental factors, such as being an only child, single-parent family structure, urban or rural residence, family size, and yearly household income, have been shown to significantly influence the proactive personality of youth (Livazović and Ham, 2019; Boele et al., 2019; Jambon and Malti, 2022). Accordingly, the control variables in this study include gender, age, educational background, only-child status, single-parent household, urban/rural location, family size, and yearly family income.

## 4 Data analysis

### 4.1 Variance of common methods

To mitigate common method bias, this study collected data across multiple time points and conducted Harman’s single-factor test using SPSS 27.0. As demonstrated in the research conducted by Podsakoff et al. (2003), it is considered acceptable for the interpretation rate of single factor cumulative variance to be less than 40% (Spector et al., 2010). The results indicate that the cumulative variance interpretation rate of the first factor is 39.813%, which is marginally lower than the critical value of 40%. Consequently, it can be deduced that there is an absence of any significant common method deviation in this study.

### 4.2 Reliability and validity test

In this study, Cronbach’s α coefficients were calculated to evaluate the internal consistency reliability of each scale. The results are presented in Table 2, demonstrate that the α coefficients of all core variables (proactive personality, parental refusal and overprotective parenting style, positive coping style, and teacher student support) range from 0.754 to 0.911. This indicates that these coefficients are greater than the acceptable standard of 0.7, with 0.900, 0.911, and 0.869 being particularly noteworthy as they exceed 0.8, suggesting excellent reliability. This finding signifies that the dosage table employed in this study exhibits high reliability.

**Table 2:** Validity analysis.

Factor	Alph
PR	0.871
PO	0.837
PP	0.900
PCS	0.911
TS	0.869

PR refers to parental rejection; PO refers to parental overprotection; PCS refers to positive coping strategies; TS teacher-student support; PP refers to proactive personality. The same applies to the table below.

Confirmatory factor analysis was performed using Amos 24.0 to evaluate the discriminant validity of the measurement model. The results, presented in Table 3, show that the six-factor model demonstrates satisfactory fit indices (*χ*^2^ = 2271.510, df = 850, *χ*^2^/df = 2.672, GFI = 0.857, RMSEA = 0.049, CFI = 0.901, TLI = 0.895, NFI = 0.851), which not only meet generally accepted criteria (MacCallum et al., 2006) but also outperform the four alternative models. This indicates good discriminant validity among the six core variables measured in this study.

**Table 3:** Confirmatory factor analysis.

Model	X^2^	df	X^2^/df	GFI	RMSEA	NFI	TLI	CFI
Five-factor model (PR; PO; PCS; TS; PP)	2271.510	850	2.672	0.857	0.049	0.851	0.895	0.901
Four-factor model (PR + PO; PCS; TS; PP)	2981.497	857	3.491	0.800	0.064	0.804	0.843	0.851
Three-factor model (PR + PO; PCS; TS + PP)	4469.493	857	5.215	0.643	0.078	0.645	0.706	0.748
Two-factor model (PR + PO + PP; PCS + TS)	7413.103	860	8.620	0.518	0.105	0.513	0.520	0.542
Single-factor model (PR + PO + PP + TS + PCS)	8399.831	860	9.767	0.477	0.112	0.448	0.447	0.473

### 4.3 Descriptive analysis

The present study employed the SPSS27.0 software for the purpose of conducting descriptive statistics and correlation analysis. The results of the analysis are presented in Table 4. As illustrated in Table 4, there is a significant positive correlation between parental rejection and positive coping strategies (*r* = 0.446, *p* < 0.01) and proactive personality (*r* = 0.297, *p* < 0.01). The present study examined the relationship between parental overprotective and proactive personality and positive coping strategies. The results showed significant negative correlations between parental overprotection and both positive coping style (*r* = −0.194, *p* < 0.01) and proactive personality (*r* = −0.148, *p* < 0.01). In contrast, positive coping strategies were positively correlated with proactive personality (*r* = 0.438, *p* < 0.01). This finding lends preliminary support to assumptions 1a and 1b.

**Table 4:** Descriptive statistics and correlation analysis.

Variable	Means	SD	PP	PR	PO	PCS	TS	G	A	O	S	Y	P	H	N	I
PP	1.76	0.428	1													
PR	1.13	0.331	0.297**	1												
PO	1.79	0.411	−0.148**	−0.526**	1											
PCS	1.85	0.357	0.438**	0.446**	−0.194**	1										
TS	2.12	0.946	0.410**	0.325**	−0.205**	0.380**	1									
G	1.14	0.347	−0.059	0.012	0.028	0.011	−0.076*	1								
A	3.06	0.857	−0.022	−0.096*	0.117**	−0.091*	0.014	0.101**	1							
O	3.03	1.967	0.028	−0.067	0.025	−0.058	−0.015	0.108**	0.06	1						
S	2.643	0.642	0.039	0.044	−0.068	−0.012	−0.028	0.028	0.025	0.213**	1					
Y	1.485	0.461	0.015	0.150**	−0.069	0.197**	0.038	0.068	−0.018	−0.016	0.115**	1				
P	3.683	0.536	0.051	0.172**	−0.03	0.023	0.016	−0.045	−0.182**	−0.270**	0.007	0.107**	1			
H	2.856	0.591	−0.024	0.087*	−0.01	0.03	−0.018	0.013	−0.039	−0.366**	−0.110**	0.152**	0.392**	1		
N	2.328	0.523	0.034	0.003	−0.003	0.003	−0.075*	0.175**	−0.015	0.506**	0.367**	−0.019	−0.092*	−0.182**	1	
I	3.707	0.54	0.038	0.121**	−0.093*	0.074	0.025	−0.051	−0.095*	−0.100**	0.085*	0.143**	0.274**	0.129**	0.029	1

G refers to Gender; A refers to age; O refers to only child; S refers to single-parent family; Y refers to respondent’s educational level; P refers to Parents’ educational level; H refers to rural or urban residence; N refers to household size; I refers to yearly household income. *p < 0.05, **p < 0.01, ***p < 0.001.

## 5 Hypothesis test

### 5.1 Examination of the mechanism through which parental rejection affects proactive personality

The results presented in Table 5 are summarized as follows. Model (1) indicates that, after controlling for all relevant variables, parental rejection is significantly and positively correlated with proactive personality (*r* = 0.254, *p* < 0.01), supporting Hypothesis 1a. Model (2) shows a significant positive correlation between parental rejection and positive coping strategies (*r* = 0.397, *p* < 0.01). Model (3) indicates that when parental rejection and positive coping strategies are both entered into the regression to predict proactive personality, positive coping strategies remain significantly and positively associated with proactive personality (*r* = 0.367, *p* < 0.01), while the association between parental rejection and proactive personality weakens (*r* = 0.108, *p* < 0.01), suggesting that positive coping strategies partially mediate this relationship and thereby supporting Hypothesis 2a. Finally, Model (4) shows that the interaction term between parental rejection and teacher-student support is significantly and positively correlated with positive coping strategies (*r* = 0.039, *p* < 0.05), confirming Hypothesis 3a.

**Table 5:** Regression results for the mechanisms linking parental rejection to proactive personality.

Variable	(1)	(2)	(3)	(4)
	PP	PCS	PP	PCS
G	−0.085*	0.000	−0.085*	0.027
A	0.024	−0.099	0.061	−0.125
O	0.047	−0.09	0.08	−0.106
S	0.016	−0.086	0.048	−0.078
Y	−0.02	0.139***	−0.071*	0.139
P	0.015	−0.054*	0.035	−0.048
H	−0.06	−0.038	−0.046	−0.018
N	0.011	0.029	0.001	0.046
I	0.001	0.006	−0.002	0.005
PR	0.254***	0.397***	0.108***	0.198***
PCS			0.367***	
TS				0.162***
PR*TS				0.039*
*R* ^2^	0.095	0.230	0.224	0.299
*F*	7.434***	20.408***	17.935***	24.210***

PR and TS in model (4) are standardized. *p < 0.05, **p < 0.01, ***p < 0.001.

Furthermore, in order to conduct additional testing of the mediating effect, this study utilised the 4.1 Process plug-in in SPSS 27.0 software and employed the bootstrapping method to execute 5,000 bootstrap samples. The findings indicated that the indirect impact of parental rejection on proactive personality through positive coping strategies was statistically significant [effect value: 0.145, standard error: 0.0209, 95% confidence interval: (0.1077, 0.1891)]. This finding lends further support to hypothesis 2a.

**Figure 2:**
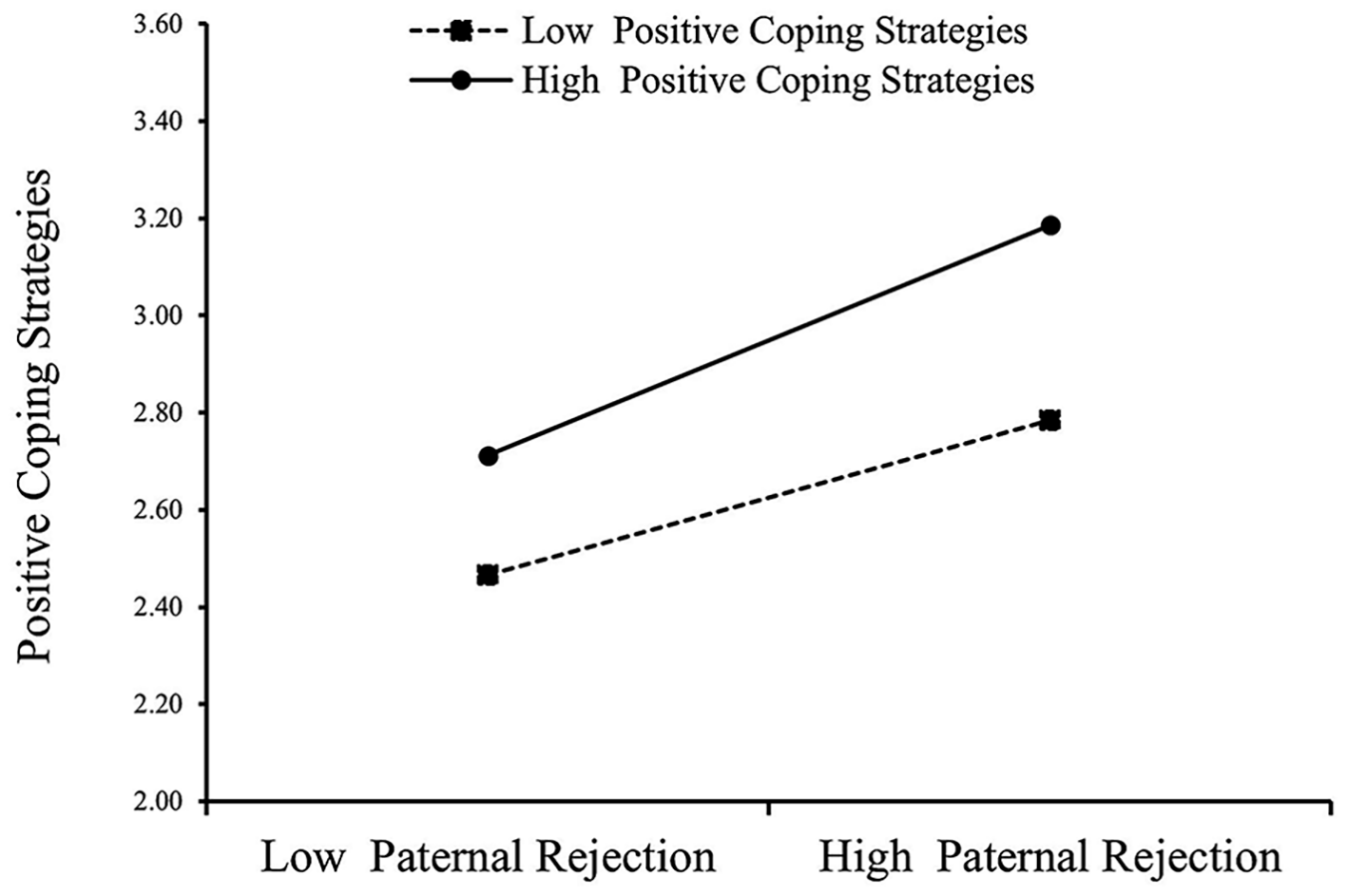
The moderating effect of teacher-student support.

To more clearly illustrate the moderating role of teacher-student support in the relationship between parental rejection and positive coping strategies, this study employs the mean plus or minus a standard deviation to distinguish the level of teacher-student support, and draws an effect diagram for the effect of interaction to make a specific analysis. As demonstrated in Figure 2, the positive effect of parental rejection on positive coping strategies is pronounced when there is a high level of support from teachers and students. In circumstances where teachers and students offer minimal support, the positive impact of parental rejection on the adoption of a positive coping style becomes weaker. This finding provides further evidence in support of hypothesis 3a.

This study further adopted Gender, only-child status, and single-parent family status as grouping variables to examine the heterogeneity of the influence of parental rejection on proactive personality. The regression results grouped by Gender showed that the regression coefficients of female and male samples were 0.273 and 0.241, both significant at the 1% level. In terms of only-child grouping, the coefficients of the only-child and non-only-child groups were 0.213 and 0.261, which were significant at the 5 and 1% levels, respectively. For the grouping of single-parent family status, the coefficients of the single-parent and non-single-parent family groups were 0.15 and 0.266, significant at the 10 and 1% levels correspondingly. To accurately distinguish inter-group differences, this study, respectively, constructed interaction terms between parental rejection and Gender, only-child status, as well as single-parent family status. Empirical results demonstrated that their corresponding coefficients were −0.02, 0.031 and 0.045, all of which were statistically insignificant. It can be concluded that the positive effect of parental rejection on proactive personality does not differ significantly across samples grouped by Gender, only-child status and single-parent family status.

### 5.2 Examination of the mechanism through which parental overprotection affects proactive personality

As shown in Table 6, after controlling for all relevant variables, Model (1) indicates a significant negative relationship between parental overprotection and proactive personality (*r* = −0.168, *p* < 0.01). This finding provides support for hypothesis 1b. Furthermore, the model (2) in the following table demonstrates that, subsequent to the control of all pertinent control variables, a significant negative correlation is present between parental overprotection and positive coping strategies (*r* = −0.222, *p* < 0.01). Based on the results from Model (3), when both parental overprotection and positive coping strategies are included in the regression equation predicting proactive personality, positive coping strategies show a significant positive relationship with proactive personality (*r* = 0.407, *p* < 0.01). while the negative association between parental overprotection and proactive personality is no longer statistically significant (*r* = −0.078, *p* > 0.05). This observation indicates that positive coping strategies function as a complete intermediary between them, thereby substantiating the validity of hypothesis 2b. Model (4) demonstrates that the interaction between parental overprotection and teacher-student support is not statistically significant. Consequently, hypothesis 3b is invalid. In addition, hypothesis 4b, which is based on hypothesis 3b, namely, the indirect effect of teacher-student support on parental overprotection on proactive personality through positive coping strategies, is also not supported.

**Table 6:** Regression results for the mechanisms linking parental overprotection to proactive personality.

Variable	(1)	(2)	(3)	(4)
	PP	PCS	PP	PCS
G	−0.075	0.014	−0.081	2.618
A	0.018	−0.115	0.065	0.041*
O	0.036	−0.109	0.08	−0.145*
S	0.014	−0.087	0.049	−0.125
Y	0.006	0.18***	−0.068*	−0.075***
P	0.038	−0.018	0.045*	0.167
H	−0.057	−0.034	−0.043	−0.022
N	0.017	0.037	0.002	−0.008
I	0.002	0.009	−0.001	0.057
PO	−0.168***	−0.222***	−0.078	0.007**
PCS			0.407***	
TS				0.215***
PO*TS				−0.032
*R* ^2^	0.031	0.083	0.216	0.209
*F*	2.214*	6.223***	17.059***	15.012***

PO and TS in model (4) are standardized. *p < 0.05, **p < 0.01, ***p < 0.001.

To further examine the mediating effect, this study applied the 4.1 Process plug-in in SPSS 27.0 software and employed the bootstrapping method to execute 5,000 bootstrap samples. The findings indicated that the indirect effect of parental overprotection on proactive personality through positive coping strategies was significant [effect value was −0.0902, standard error was 0.0218, 95% confidence interval was (−0.1347, −0.0498)]. This finding provides further evidence in support of hypothesis 2b.

Further, this study adopted demographic factors including Gender, only-child status, and single-parent family status to explore the differential effects of parental overprotection on proactive personality. Regression analysis grouped by Gender showed that parental overprotection had no significant effect on proactive personality in the female group (*r* = −0.097, *p* > 0.5), whereas it exerted a significant negative effect in the male group (*r* = −0.18, *p* < 0.01). This finding can be attributed to differences in traditional social gender roles. Society expects females to be gentle and reserved with low demands for independent exploration, and parental overprotection conforms to female role expectations, thus having no significant influence on their proactive personality. In contrast, males are socially expected to be independent and responsible. Parental overprotection runs counter to males’ developmental expectations and restricts their autonomous exploration, thereby significantly inhibiting males’ proactive personality (Yao et al., 2022). Regression results grouped by only-child status indicated that parental overprotection had no significant impact on proactive personality among only children (*r* = −0.174, *p* > 0.5), while a significant negative effect emerged in the non-only-child group (*r* = −0.173, *p* < 0.01). From the perspective of family rearing, only children have long lived in an environment of intensive family care and overprotection and become highly adapted to such parenting patterns. Accordingly, parental overprotection fails to exert a significant effect on their proactive personality. Non-only children can obtain opportunities for independent practice and exploratory growth through daily sibling interactions, while parental overprotection limits their independent development space and hinders the formation of proactive traits (Rong et al., 2022), hence producing a significant negative effect on their proactive personality. In terms of grouping by single-parent family status, parental overprotection had no significant effect on proactive personality in the single-parent family group (*r* = −0.185, *p* > 0.5), but exerted a significant negative effect in the non-single-parent family group (*r* = −0.166, *p* < 0.01). Single-parent families have unique family structures and parenting modes, and individuals from such families develop strong adaptability to family rearing styles, rendering parental overprotection unable to significantly influence their proactive personality. Adolescents from non-single-parent families grow up in a stable and complete environment with natural space for autonomous exploration and independent development. Parental overprotection restricts the cultivation of their independent and proactive traits, thereby significantly negatively predicting proactive personality (Ryu et al., 2025).

### 5.3 Moderated mediation test

Furthermore, as shown in Table 7, this study utilised the 4.1 plug-in of SPSS 27.0 software to estimate the confidence interval of the indirect effects and differences of positive coping strategies on parental rejection and proactive personality formation under conditions of high and low support from teachers and students. The 95% confidence interval of low teacher-student support was found to be [0.0373, 0.0833], the 95% confidence interval of high teacher-student support was [0.0621, 0.1148], and the 95% confidence interval of the indirect effect difference between high and low conditions was [0.0621, 0.1148] [0.0621, 0.1148]. It was established that these intervals did not include 0, thus confirming the hypothesis that teacher-student support positively moderate the mediating role of positive coping strategies in the relationship between parental rejection and proactive personality shaping. However, because teacher-student support did not show a moderating effect between parental overprotection and positive coping strategies, the moderated mediation effect was not established in this pathway; consequently, the results of this test are not reported in the study’s findings.

**Table 7:** Analysis results of moderated mediation effects.

Teacher-student support	Indirect effect	Standard error	95% confidence interval
Low TS	0.0582	0.0117	[0.0373,0.0833]
High TS	0.0872	0.0135	[0.0621,0.1148]
Difference in the indirect effects between high and low conditions	0.0145	0.0057	[0.0621,0.1148]

Low teacher-student support is the MEAN-1SD, while high teacher-student support is the MEAN + 1SD.

## 6 Discussion and conclusion

Based on ecological systems theory, this study aimed to investigate how parental negative parenting styles influence young adults’ proactive personality, focusing on the mediating effect of positive coping strategies and the moderating role of teacher-student support. The main findings are summarized as follows:

Firstly, the present study discloses that two distinct forms of negative parenting styles exert disparate influence on the proactive personality of young adults. The present study challenges the prevailing perspective that negative parenting is typically detrimental (Shelton and Harold, 2008; Xu and Yan, 2023). Rather, it posits that the parental rejection experienced by young adults exerts a favourable predictive influence on the development of an proactive personality. This finding can be understood from multiple perspectives.

From the perspective of family systems theory, the positive predictive effect of parental rejection and the negative predictive effect of parental overprotection may reflect the high degree of coordination in parenting behaviors within Chinese families (Xie et al., 2021). The mother–child and father-child dyads, as core subsystems, tend to maintain consistency in their parenting climate, leading young adults to perceive negative parenting as a unified family psychological environment (Yang et al., 2016). However, this overall family effect may also obscure the functional differences between paternal and maternal roles. The positive association of parental rejection found in this study may, in fact, arise because the challenge posed by paternal rejection to young adults’ independence activates a motivation for “adversity-driven growth” which partially or fully offsets the emotional harm potentially caused by maternal rejection (Peng et al., 2024). Similarly, the strong negative effect of parental overprotection may stem from its dual suppression of both the paternal activation function and the maternal emotional support capacity (Van der Giessen and Bögels, 2018).

From a macro-cultural and institutional perspective, this positive effect is deeply embedded in China’s specific social ecology. Education is widely regarded as the core pathway to social mobility and family honor, and parents hold an extremely high willingness to invest in their children’s education, along with correspondingly high expectations of return (Liu, 2012). This cultural consensus, akin to the saying “hating iron for not becoming steel” (expressing frustrated but caring, high expectations), makes parental rejection more likely to be interpreted as spurring rather than harming. At the same time, China’s highly stratified and merit-selective higher education system turns academic achievement into the hard currency for gaining social recognition (Liu, 2025), while the interdependent self-construal prevalent among young people predisposes them to restore threatened family bonds through social recognition (Chen et al., 2022).

From the perspective of the adaptation process, this transformation involves a shift of multiple resources from the family system to the school system. The process may begin with the emotional distress and damaged self-esteem triggered by family rejection, a state of cognitive disequilibrium that becomes a driving force for individuals to seek change and turn to alternative resources within the school context (Zhao et al., 2023). During this transition, peer acceptance compensates for the lack of family support and provides social capital for individuals to “disembed” from family risks (Tung and Lee, 2014); teacher autonomy support not only buffers the direct impact of high-risk family environments on problem behaviors but also attenuates the negative effects of deviant peer mechanisms on individual adaptation (Cui and Bao, 2025). Thus, through interactions within the multi-ecological system composed of family, peers, and teachers, individuals gradually build self-efficacy in actively coping with the external environment and establish stable behavioral patterns.

This study further examined the heterogeneity of the above effects by child gender. The results showed that the positive effect of parental rejection was consistently present in both males and females, suggesting that the “adversity-activated proactivity” mechanism may be universal across genders. In contrast, the negative effect of parental overprotection exhibited distinct gender differentiation: It was significant only in males. This difference can be understood by integrating social role expectations and existing empirical evidence. From the perspective of socialization, independence and exploration are more strongly expected of males; overprotection thus creates a more severe conflict with males’ developmental needs for autonomy, thereby inhibiting their proactive personality more significantly. This is consistent with previous findings that negative parenting uniquely influences boys’ externalizing behavior problems or proactive aggression (Wang, 2017; Lent and Murray-Close, 2022). Females, on the other hand, may have a higher tolerance for overprotection due to social expectations that do not emphasize autonomy as strongly, thereby attenuating the negative impact on their proactive personality. These findings suggest that future research should incorporate the interaction between parent and child gender into models to reveal gendered family interaction effects.

It is important to emphasize that the positive association between parental rejection and a proactive personality found in this study does not imply that rejecting parenting is desirable or beneficial. Rather, this effect reflects a “stress-activated compensation” psychological mechanism. That is, when individuals experience emotional deprivation at home, they are compelled to seek developmental resources and psychological fulfillment outside the family system. This process entails psychological costs, such as emotional distress and fluctuating self-esteem, and does not constitute a healthy developmental pathway. Furthermore, this effect is embedded within a specific Chinese cultural context, such as the highly instrumentalized nature of education and family expectations closely tied to academic achievement, and it should not be generalized to other cultural settings. Therefore, the present findings should not be interpreted as “rejection is beneficial,” but rather as a compensatory, adaptive response to a negative parenting environment under specific cultural and psychological conditions.

Second, this study clarified the mediating role of positive coping strategies in the relationship between negative parenting styles and proactive personality. The findings indicate a partial mediating effect, suggesting that parental rejection may foster proactive personality in youths by encouraging them to adopt more positive coping strategies. Concurrently, parental overprotection systematically hinders the acquisition and application of such strategies. This occurs through the weakening of individuals’ opportunities for independent exploration and the reduction of self-efficacy. The decline in self-efficacy further undermines the capacity or willingness for positive coping, which, in turn, indirectly hinders the development of a proactive personality in young adults. This finding not only validates the compensatory pathway of “adversity-induced agency” (Sarkar et al., 2015; Lu et al., 2019), but also reveals, from a positive youth development perspective, that young people’s proactive personality is an actively constructed process that can be systematically supported. Specifically, the challenging situations created by rejecting parenting may unexpectedly activate individuals’ ability to draw upon their own developmental assets. Teacher and peer support, as key external developmental assets, further reinforces this positive transformative pathway (Lerner et al., 2021). Consequently, young people are not passive victims, but rather agentic subjects who actively construct their own development through interactions across multiple systems.

Thirdly, this study reveals the boundary conditions of the moderating role of teacher-student support. The results show that such support effectively moderates the negative effects of parental rejection on positive coping, but its moderating effect on parental overprotection is not significant. This difference stems from the distinct mechanisms of the two parenting styles. Parental rejection primarily harms individuals by damaging their perception of external support and emotional security. In this context, high-quality teacher–student support serves as a compensatory external resource, directly compensating for the lack of family support, thereby weakening the negative impact of rejection and providing the necessary socio-emotional foundation for the development of positive coping strategies (Kang et al., 2024). This finding is consistent with existing research: emotional conflict in teacher-student relationships significantly moderates learning motivation, whereas pure emotional support does not necessarily have a significant effect (Li C. et al., 2022; Li J. et al., 2022), indicating that external support primarily compensates for ‘relational connection’ and ‘emotional security’ rather than internal autonomy (Virat, 2022). In contrast, parental overprotection erodes the healthy development of internal motivational systems, such as autonomy and self-efficacy (Cui et al., 2019; Feng et al., 2024). This parenting style results in a weak sense of responsibility and autonomous drive, representing a more internalized psychological developmental issue. Therefore, when the damage is concentrated on interpersonal security and the perception of external support, teacher-student support can effectively compensate; when the damage is concentrated on internal autonomy and self-efficacy, more targeted interventions such as autonomy support and skill-building exercises are needed (Hughes et al., 2014).

### 6.1 Theoretical contribution

By clarifying the different influence paths of different negative parenting styles, this study has made the following three important contributions to the related theoretical fields:

Firstly, this study deepens and refines the theoretical cognition about the impact of family environment on individual development. The majority of extant studies regard negative parenting as a homogeneous risk factor. However, this study demonstrates that there are significant differences in the influence mechanism of rejection and overprotection on young adult’s proactive personality, and that the direction of action is even opposite. This finding compensates for the limitations of extant literature on the absence of attention to the internal heterogeneity of negative parenting, demonstrates the theoretical value of typological differentiation, and provides evidence for understanding the complexity of family function.

Secondly, the study elucidates the differentiation path of positive coping strategies under two types of negative parenting styles, and clarifies the internal psychological mechanism between family environment and personality development. The study found that positive coping strategies played two different mediating roles in this process. In the context of parental rejection, the compensatory ability of individuals to actively develop in response to emotional loss is more pronounced. Conversely, in the context of parental overprotection, the absence of fully developed psychological skills is attributable to the deprivation of opportunities for independent exploration. This fundamental distinction naturally links family education with individual psychological development.

Finally, this study contributes to the development of ecological systems theory by offering insights into the differential moderating role of teacher-student support. The research confirms that the interaction between family and school is not a simple superposition, but rather a profound mechanism of matching. The efficacy of external support is contingent upon its ability to compensate for the psychological resources that have been compromised by the particular family environment. The “match compensation” perspective provides a novel theoretical entry point for understanding how multiple microsystems affect individual development, and defines the effectiveness and boundary conditions of the interaction within the system.

### 6.2 Enlightenment

In consideration of the research conclusions outlined above, this study offers the following management implications for the education and developmental intervention practices concerning youth mental health:

Firstly, it has been determined that there is a clear boundary for the buffer effect of external support. Schools and related educational institutions can therefore promote the construction of more targeted student support paths. The findings of this study indicate that the unified intervention model may exert a negligible effect, which necessitates differentiation according to the type of family upbringing experienced by students. Consequently, it is recommended that educators pay close attention to assessing students’ family environments and provide differentiated guidance accordingly. For instance, students who perceive familial emotional rejection may benefit from the provision of structured group activities, which have been demonstrated to engender a sense of emotional security and belonging (Byrom, 2018; Yao et al., 2023). Furthermore, the establishment of supportive tutor relationships has been shown to have a positive impact on students’ well-being (Glazzard et al., 2021; Mazzer and Rickwood, 2015). Conversely, students who are raised in overprotective environments may require opportunities for independent exploration and the activation of intrinsic motivation to facilitate positive development (Eccles et al., 1991; Geller and Boehm, 2020). This signifies an enhanced distribution of psychological support resources throughout the “assessment understanding response” chain.

Secondly, it is recommended that parents focus on refining their parenting style and cultivating positive and healthy interactions with their children. The present study reveals that the two types of negative parenting styles, namely “rejection” and “overprotection,” have different influence paths on children’s development. Consequently, targeted classified guidance should be implemented within the framework of parent education and guidance. For parents who have become accustomed to a parenting style characterised by rejection, it is imperative to facilitate their realisation of the significance of emotional response and warm acceptance. Furthermore, it is crucial to encourage them to rectify the parent–child relationship by fostering companionship, demonstrating active listening, and expressing recognition (Bowes et al., 2010; Luo et al., 2020). It is imperative that we assist parents who exhibit overprotective tendencies in their parenting style. We must enlighten them on the significance of autonomy in the developmental process of young adults. It is crucial to guide them in the process of gradually relinquishing control, authorising their children to make independent decisions within the confines of safety. This approach aims to transition the paradigm of parenting from an approach of “alternative outsourcing” to a model of “support empowerment” (Van Petegem et al., 2012; Beyers et al., 2025).

### 6.3 Limitations and future research

The limitations of this study are mainly reflected in the following aspects. First, the study employed a cross-sectional design. While this approach reveals correlational relationships among variables and the theoretically proposed mediating and moderating pathways, it cannot rigorously establish causal temporal sequences or capture the dynamic developmental processes of individuals. For instance, there may exist reciprocal relationships among parenting styles, positive coping, and proactive personality, in which these factors mutually influence and shape one another in a cyclical manner.

Second, the core variables in this study were all measured using self-report methods, which may introduce common method bias and social desirability bias. For example, participants may have tended to overreport positive coping strategies or underreport negative family experiences due to social expectations, and retrospective reports of parenting styles may also have been affected by recall bias. Future research could incorporate multiple reporters (e.g., parent self-reports, teacher ratings) and multiple methods (e.g., observation, interviews) to obtain more objective measurement indicators and cross-validate the findings of this study.

Furthermore, the study sample was primarily drawn from the southwestern ethnic minority region of China, an area with distinctive sociocultural and economic characteristics. This specificity may limit the external validity of the findings when extended to other regions, particularly populations from different cultural backgrounds. In addition, this study measured parenting style as an overall variable and was unable to separately examine the unique effects of fathers and mothers. As discussed, paternal rejection and maternal rejection may differ in their psychological meanings. Future research could measure paternal and maternal parenting behaviors separately in order to more precisely elucidate the differential roles of fathers and mothers in the formation of proactive personality.

Finally, the research model focused primarily on the individual and family levels and did not fully consider or control for important school and peer contextual variables, such as different styles of teacher management, varying classroom climates, and the closeness of peer relationships. These factors may directly or indirectly influence the psychosocial functioning of young people, and neglecting them could lead to biased estimates of the family influence pathways. Future research could employ longitudinal designs to test the generalizability of the model across broader geographical and cultural samples and integrate meso-system variables such as school and peer factors to build a more comprehensive ecological model. In addition, combining multi-source data with qualitative methods would allow for deeper exploration of nonlinear relationships and interaction effects among the variables.

## Data Availability

The datasets presented in this study can be found in online repositories. The names of the repository/repositories and accession number(s) can be found in the article/Supplementary material.
