# Supplementary material for: Is negative parenting always harmful? The differential effects of parental rejection and parental overprotection on proactive personality in young adults
Source: Front Psychol. 2026 May 28;17:1798297. doi: 10.3389/fpsyg.2026.1798297 (PMC13253630; doi:10.3389/fpsyg.2026.1798297)
Supplement: Supplementary file 1 [file Supplementary_file_1.DOCX]

**Dear Student,**

Greetings! Thank you for taking the time to participate in this survey. Our research team is currently investigating the influence of parenting styles on adolescents’ growth mindset. The survey is conducted anonymously and will take approximately 5-7 minutes to complete. Your honest opinions and experiences will provide invaluable support to our research. We sincerely appreciate your participation and cooperation! Rest assured that all information will be kept strictly confidential and used solely for academic purposes.

**Part 1: Personal and Family Background Information**

1. Gender

Male

Female

1. Age

18-21

22-25

1. Only Child

Yes

No

1. Single-Parent Household

Yes

No

1. Your Education Level

Junior high school or below

High school (including vocational secondary school)

Associate degree (including night university)

Bachelor’s degree

Graduate degree (Master’s/PhD)

1. Parents’ Education Level

Primary school or below

Junior high school

High school (including vocational secondary school)

Associate degree (including night university)

Bachelor’s degree

Graduate degree (Master’s/PhD)

1. Household Registration Type

Rural

Urban

1. Number of Family Members

2 or fewer

3

4

5 or more

1. Annual Household Income

¥20,000 or less

¥20,001–30,000

¥30,001–40,000

¥40,001–50,000

¥50,001–60,000

¥60,001–70,000

¥80,001 or more

**Part 2: Simplified Parenting Style Questionnaire** (Please select the most appropriate option for each item based on your actual experience: 1=Never, 2=Occasionally, 3=Frequently, 4=Always. Note: Each question has separate options for father and mother. If you grew up in a single-parent household or lost a parent, you may answer only the relevant section. Please ensure all items are completed.)

1. My [father/mother] often loses temper with me without explaining why.

1 2 3 4

1. My [father/mother] praises me.

1 2 3 4

1. I wish my [father/mother] wouldn’t worry excessively about what I’m doing.

1 2 3 4

1. My [father/mother]’s punishments are harsher than I deserve.

1 2 3 4

1. My [father/mother] demands I report everything I do outside the home.

1 2 3 4

1. I feel my [father/mother] tries to make my adolescence meaningful and enriching.

1 2 3 4

1. My [father/mother] criticizes me as "lazy" or "useless" in front of others.

1 2 3 4

1. My [father/mother] forbids me from activities other kids are allowed, fearing I’ll get hurt.

1 2 3 4

1. My [father/mother] consistently encourages me to excel.

1 2 3 4

1. I feel my [father/mother]’s worry about potential harm to me is exaggerated.

1 2 3 4

1. When upset, I feel comforted by my [father/mother]’s encouragement.

1 2 3 4

1. I’m often treated as the "scapegoat" or "black sheep" at home.

1 2 3 4

1. I sense my [father/mother]’s affection through their words/expressions.

1 2 3 4

1. My [father/mother] often embarrasses me in front of others.

1 2 3 4

1. My [father/mother] allows me to go places I like without excessive worry.

1 2 3 4

1. I feel my [father/mother] interferes with everything I do.

1 2 3 4

1. I share a warm, caring bond with my [father/mother].

1 2 3 4

1. My [father/mother] imposes rigid rules without flexibility.

1 2 3 4

1. My [father/mother] punishes me even for minor mistakes.

1 2 3 4

1. My [father/mother] controls what I wear or how I look.

1 2 3 4

1. When I succeed, I feel my [father/mother] is proud of me.

1 2 3 4

**Part 3: Proactive Personality Scale** (Please select the option that best reflects your situation: 1=Strongly Disagree, 2=Disagree, 3=Uncertain, 4=Agree, 5=Strongly Agree.)

1. I actively seek new ways to improve my life.

1 2 3 4 5

1. Wherever I am, I proactively drive constructive change.

1 2 3 4 5

1. Seeing my ideas materialize excites me the most.

1 2 3 4 5

1. When encountering undesirable situations, I take initiative to resolve them.

1 2 3 4 5

1. Regardless of success probability, I persistently work to realize my beliefs.

1 2 3 4 5

1. I stand by my ideas even when facing opposition.

1 2 3 4 5

1. I excel at identifying opportunities.

1 2 3 4 5

1. I consistently pursue more effective approaches.

1 2 3 4 5

1. No obstacle can deter me from actualizing ideas I believe in.

1 2 3 4 5

1. I detect promising opportunities earlier than others.

1 2 3 4 5

**Part 4: Simplified Coping Style Questionnaire** (Please select the option that best matches your actual behavior: 1=Never Use, 2=Occasionally Use, 3=Sometimes Use, 4=Frequently Use)

1. I distract myself from troubles through work, study, or other activities.

1 2 3 4

1. I talk to others to share my inner worries.

1 2 3 4

1. I try to focus on the positive aspects of situations.

1 2 3 4

1. I reevaluate my priorities to rediscover what matters in life.

1 2 3 4

1. I avoid catastrophizing problems.

1 2 3 4

1. I stand my ground and fight for what I want.

1 2 3 4

1. I brainstorm multiple solutions to problems.

1 2 3 4

1. I seek advice from relatives, friends, or classmates.

1 2 3 4

1. I modify my behaviors or correct personal shortcomings.

1 2 3 4

1. I learn from others' approaches to similar difficulties.

1 2 3 4

1. I engage in hobbies or recreational activities to relieve stress.

1 2 3 4

1. I consciously suppress feelings of disappointment, regret, sadness, or anger.

1 2 3 4

1. I take breaks or vacations to temporarily avoid problems.

1 2 3 4

1. I use smoking, alcohol, medication, or overeating to cope.

1 2 3 4

1. I believe time will resolve things and just wait passively.

1 2 3 4

1. I try to completely forget about the issue.

1 2 3 4

1. I rely on others to solve problems for me.

1 2 3 4

1. I resign myself to reality when no alternatives exist.

1 2 3 4

1. I fantasize about miraculous solutions.

1 2 3 4

1. I console myself through self-talk.

1 2 3 4
